# Using GPS Technology to Quantify Human Mobility, Dynamic Contacts and Infectious Disease Dynamics in a Resource-Poor Urban Environment

**DOI:** 10.1371/journal.pone.0058802

**Published:** 2013-04-08

**Authors:** Gonzalo M. Vazquez-Prokopec, Donal Bisanzio, Steven T. Stoddard, Valerie Paz-Soldan, Amy C. Morrison, John P. Elder, Jhon Ramirez-Paredes, Eric S. Halsey, Tadeusz J. Kochel, Thomas W. Scott, Uriel Kitron

**Affiliations:** 1 Department of Environmental Studies, Emory University, Atlanta, Georgia, United States of America; 2 Fogarty International Center, National Institutes of Health, Bethesda, Maryland, United States of America; 3 Department of Entomology, University of California Davis, Davis, California, United States of America; 4 Department of Global Health Systems and Development, Tulane University School of Public Health and Tropical Medicine, New Orleans, Louisiana, United States of America; 5 Graduate School of Public Health, San Diego State University, San Diego, California, United States of America; 6 U.S. Naval Medical Research Unit, Lima and Iquitos, Peru; 7 Naval Medical Research Center, Silver Spring, Maryland, United States of America; INSERM & Universite Pierre et Marie Curie, France

## Abstract

Empiric quantification of human mobility patterns is paramount for better urban planning, understanding social network structure and responding to infectious disease threats, especially in light of rapid growth in urbanization and globalization. This need is of particular relevance for developing countries, since they host the majority of the global urban population and are disproportionally affected by the burden of disease. We used Global Positioning System (GPS) data-loggers to track the fine-scale (within city) mobility patterns of 582 residents from two neighborhoods from the city of Iquitos, Peru. We used ∼2.3 million GPS data-points to quantify age-specific mobility parameters and dynamic co-location networks among all tracked individuals. Geographic space significantly affected human mobility, giving rise to highly local mobility kernels. Most (∼80%) movements occurred within 1 km of an individual’s home. Potential hourly contacts among individuals were highly irregular and temporally unstructured. Only up to 38% of the tracked participants showed a regular and predictable mobility routine, a sharp contrast to the situation in the developed world. As a case study, we quantified the impact of spatially and temporally unstructured routines on the dynamics of transmission of an influenza-like pathogen within an Iquitos neighborhood. Temporally unstructured daily routines (e.g., not dominated by a single location, such as a workplace, where an individual repeatedly spent significant amount of time) increased an epidemic’s final size and effective reproduction number by 20% in comparison to scenarios modeling temporally structured contacts. Our findings provide a mechanistic description of the basic rules that shape human mobility within a resource-poor urban center, and contribute to the understanding of the role of fine-scale patterns of individual movement and co-location in infectious disease dynamics. More generally, this study emphasizes the need for careful consideration of human social interactions when designing infectious disease mitigation strategies, particularly within resource-poor urban environments.

## Introduction

Routine movements of individuals within cities are of paramount importance for planning urban infrastructures [1], developing transport and commuting alternatives [1], [2], improving wireless communication networks [3], [4], promoting healthy lifestyles [5], and preventing or responding to emergence, propagation and persistence of infectious disease [6]–[11]. People routinely engage in activities that vary in relative frequency and duration as well as in geographic location and, more importantly, their spatial behavior can be affected by changes in social and economic contexts [12]. Understanding the statistical patterns that characterize human mobility within cities poses fascinating scientific questions and major methodological, technical and ethical challenges [13], [14], particularly when aiming at understanding their role in spatio-temporal human-mediated processes such as infectious disease transmission [6]–[11].

Early mathematical models of infectious diseases assumed individuals as having an equal chance of transmitting and getting exposed to disease agents (i.e., homogenous mixing), ignoring stochastic variations in transmission potential or heterogeneities in contact patterns [15]. Empirical evidence shows that contact rates are indeed highly heterogeneous [10], [16], [17], in part owing to the complex and dynamic fabric of human social relationships [10], [18]. Therefore, individual social structure and movement patterns play a significant role in modulating contact rates, affecting the transmission, spread and persistence of pathogens and drug resistance [10], [11]. Inferring infectious disease contact structures from human mobility requires the explicit consideration of the spatial and temporal dimensions of pathogen transmission, which are contingent on the type of pathogen and its mode of transmission [15]. Most mathematical models of directly transmitted pathogens assume that contacts are fixed (edges in a contact network do not change over time or during the duration of an outbreak). In reality, human movement and potential infectious contacts are highly dynamic, and theoretical models have shown that such heterogeneity can have profound impacts on the transmission and stability of disease outbreaks [19]–[21]. In fact, accounting for human commuting behaviors in meta-population models (by simulating working-age individuals’ daily return to their home census district), significantly reduced the speed of propagation and the predicted impact of disease epidemics in comparison to models assuming irregular (probabilistic or random) movements [11], [20], [22], [23].

The recent availability of location-aware technologies such as mobile phones, GPS-enabled devices, wireless local area networks and personal digital assistants has provided quantitative evidence that human spatial behavior is recursive and dominated by highly reproducible scaling laws [4], [18], [24]–[26]. Individuals tend to visit a few locations, where they spend the majority of their time, and the availability of transportation and commuting alternatives facilitates their movement across multiple spatial and temporal scales [13]. Mobile-phone data have been the most widely used technology to capture and describe human mobility within cities. Because most information derived from mobile phones is coarsely tagged at scales ranging from hundreds to thousands of meters (depending on antenna configuration and availability of GPS-enhanced positioning) and sparsely collected over time (mobile phone data provides information of the location where calls occurred, meaning that only a few data points are located over a single day), information about the rich and complex repertoire of fine scale movement patterns and spatial behaviors of individuals within cities remains elusive.

Most of our understanding of human mobility and spatial behavior within cities is based on research performed in developed societies (e.g., [1], [2], [27], [28]). This translates into a critical knowledge gap because approximately 70% of the ∼3.3 billion people comprising the global urban population live in resource-poor urban environments [29], for which limited information on human movement patterns and urban infrastructure exist (see [30] for an example of mobility quantification at coarser scales within an African country). This near absence of detailed studies of within-city movements in developing countries is associated with the challenges of obtaining reliable mobility information for large segments of the population. In addition to the limited access to data from cell-phone carriers, issues of antenna density, phone ownership and available technologies (e.g., a scarcity of GPS-enabled systems) severely affect data quality and accuracy. Acquiring detailed movement information is of significant interest for the development of more accurate mechanistic models quantifying, for instance, the impacts of mobility on infectious disease dynamics and the outcome of disease mitigation strategies.

Unmanaged planning, limited public infrastructure and informal employment and economies characterize most urban environments in developing countries [31], potentially making them more socially and environmentally complex than their developed counterparts. We hypothesize that human mobility and spatial behavior within resource-poor urban environments are strongly modulated by geographic distance (in opposition to developed countries, where transportation networks and high vehicle ownership facilitate long distance movements) and that highly informal economic and social structures contribute to the emergence of unstructured daily routines, a pattern that can have significant impacts in spatio-temporal human-mediated processes such as infectious disease transmission. Here, we report our application of GPS technology to quantify fine scale human mobility parameters and the development of highly detailed individual-based simulation models for the quantification of the impact of fine-scale mobility (within a city neighborhood) in infectious disease dynamics.

## Materials and Methods

Specific details about devices used, subject pool, data analysis and simulation model parameters are provided in detail in the Supplementary Material (File S1), whereas additional figures and tables are presented in File S2.

### Study area

The Amazon city of Iquitos (73.2°W, 3.7°S, 120°m above sea level) in the Department of Loreto, north-eastern Peru (Figure S1 in File S2), is a geographically isolated resource-poor urban environment of approximately 370,000 inhabitants. Iquitos has a high population density (most of its inhabitants live in an urban area of ∼30 sq. km [32]), a highly informal and dynamic economic structure (33.4% of the economically active population is either unemployed or informally employed [32]), and diverse modes of formal and informal public transportation (personal motorcycles, ∼20,000 auto rickshaws, a few bus lines, and small to large size boats are used as modes of transportation). The major industries in the area are small commercial enterprises, fishing, oil, lumber, tourism and, to a lesser extent, agriculture.

### Study Design

#### Human Subjects Approval

Before enrolling in our study, each participant was provided with detailed information about the type of data collected with GPS (and descriptions about the GPS technology itself) and how such data would be used within the context of the study. Focus group discussions with representative segments of the Iquitos population resulted in an information pamphlet addressing concerns and questions related to the GPS technology [33]. Participants were given a 24–48 hour period to decide whether to participate or not in the study. For children, verbal assent of the minor and written consent of the parent or caretaker were required, whereas for adults, a written consent was required. After GPS data collection, a strict protocol for storage (in a secure MySQL database) and management was followed. The procedures for enrolment of participants and GPS data management were approved by the Institutional Review Boards of University of California, Davis (2007.15244); Naval Medical Research Center Detachment (NMRCD 2007.0007), which included Peruvian representation; and Emory University (IRB9162).

#### Tracking human movements

GPS-data-loggers (“Igot-U GT120”, Mobile Action Technology Inc.) were used to continuously track individual movement patterns of Iquitos residents for a two week period. GPS accuracy (point and line accuracy of 4.4 m and 10.3 m, respectively), acceptance by participants, and deployment were described previously [33], [34]. GPS units were programmed at a 2.5 min collection frequency interval and set to turn off at night (from midnight to 06∶00 AM). Given the logistic limitations encountered when tracking large numbers of individuals simultaneously, which could affect the quality of data collected, we tracked small groups of individuals from two defined neighborhoods continuously for 15 days in order to capture their average fine-scale spatial routine. Most (77%) GPS tracking of school age children was performed during the period when schools were in session.

**Figure 1:**
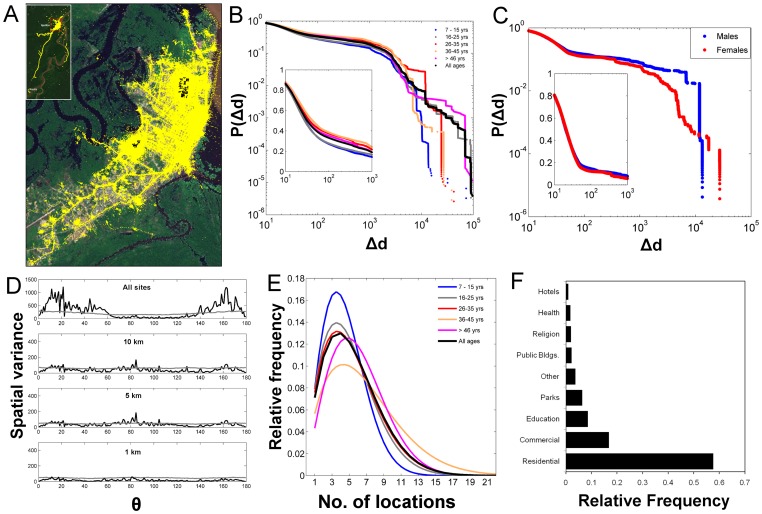
Mobility parameters inferred from GPS data-logger data. (A) Raw GPS locations (∼2.3 million points) obtained from tracking the movements of 582 individuals. Map inset shows out-of-city movements. (B) Human movement kernels (probability of movement outside an individual’s home (*P(Δd)*) for all individuals and for different age groups. Inset in frame B shows the probability of movement within 1 km from an individual’s home. (C) *P(Δd)* for males and females. (D) Spatial wavelet variance (black line) as a function of the angle from a person’s home (*θ*). Anisotropy is detected when variance is higher than the randomness expectation (grey line). (E) The probability distribution of the number of places an individual routinely visited. (F) The relative frequency of visitation across type of places.

The analyzed trajectories from 582 participants (see “subject pool” section in File S1 and Table S1 in File S2 for details on participant pool) included 2,299,718 raw GPS positions tagged with date, time, elevation, latitude and longitude (Figure 1A). A data reduction algorithm that aggregates consecutive GPS readings located within pre-specified spatial and temporal windows was used to identify the geographic position and total time a participant spent at a given place. This algorithm (named I-cluster) aggregates GPS readings that are within a spatial (*d*) and temporal (*t*) window and estimates the total time a participant spent within such spatio-temporal buffer [35]. Gaps in the GPS data associated with an I-cluster identified place can emerge due to signal loss or an individual leaving the place and returning *t* minutes later (i.e., intermittent visits). The I-cluster algorithm uses a threshold time (*tintv*  = 30 min) to separate between data gap types [35] (*tintv*<30 minutes indicated a transient loss of GPS signal and *tintv*>30 minutes indicated a participant revisited the location *t* minutes after the first visit). Refer to [35] for a detailed description and code of the I-cluster algorithm. Based on the inherent error of GPS data, we set the algorithm’s parameters as *d* = 20 m and *t*  = 15 min [34].

The lot code and land-use description (residential, commercial, recreational, health, religion, others) of the places visited by each participant were determined by joining the I-cluster data with our highly detailed and frequently updated Geographic Information System (GIS) of the city of Iquitos [36]. For each identified place, the total time of permanence (in minutes) and the total time and frequency of visits were registered. The temporal patterns of visitation to each I-cluster identified place were assessed by determining, from the raw GPS locations, the day of the week and time of the day each visit occurred. Independent analyses of simultaneous GPS and Semi Structured Interview mobility data from 101 Iquitos residents show that, of 1,455 identified locations, 11.3% were concordant between methods, whereas 65.8% were identified only by interviews and 22.8% by the I-cluster algorithm only (Paz-Soldan et al. unpublished data). As reported in a systematic review, the rates of GPS data loss or mismatch could emerge from signal drop-outs, dead batteries, participants’ not wearing the units, signal loss during the initialization period or misuse of the device [28]. To minimize this issue, we: (1) individually explored the raw GPS data from each participant for places not identified by the I-cluster algorithm (i.e., not statically visited by participants, like parks or markets, or places not consistently identified by the algorithm); (2) analysed the raw GPS locations by applying a 20 m buffer around each I-cluster identified place, and (3) performed analyses on aggregated data (by age group or sex) to avoid any potential bias emerging from the description of movement behaviors of specific individuals. In the context of this study, we consider *locations* as the raw GPS points and *places* as the locales identified by the data reduction algorithm.

#### Quantifying human movement parameters

Maximum likelihood methods were applied to the raw GPS locations to fit several mathematical distributions (truncated power-law, linear decay or exponential decay) to the cumulative density function of the distance to each participant’s home. Such movement kernels were inferred for all individuals and each sex separately as well as for different age groups: 7–15 years, 16–25 years, 26–35 years, 36–45 years, 46 years and older. Age categories were defined using 10-year bins because the limited number of tracked individuals precluded the generation of narrower age groups. To determine if the distribution of visited places occurred predominantly in a given cardinal angular direction (*θ*) we implemented a spatial wavelet analysis. A wavelet function *g*(*x*) is a scalable windowing function. In our study we used the French Top Hat [37] as a wavelet function. The main metric derived from fitting the wavelet function to the data is the wavelet positional variance. Peaks in this variance indicate directions from each individual’s home where most of the visited places are located. In order to separate true patterns from random fluctuations, the significance of the wavelet analysis was determined by comparing the observed variance with the one obtained from 999 Monte Carlo simulations [37]. The analysis was performed for all places, and for places located within 10 km, 5km and 1 km of an individual’s home.

#### Inferring mobility networks

GPS tracking was not simultaneous for all participants, which were mainly tracked within a 1-year period (see “Subject Pool” section in File S1). We considered the 15-day tracking period sufficient to characterize participant’s short-term spatial routine (our study did not consider places visited at frequencies smaller than 1/15 days or changes in movement that may occur over longer time periods), and collapsed the movement data to generate a “static” undirected bipartite graph (*N_ij_*), representing all participants (*i*) linked to the places (*j*) they visited (see Table 1 for a complete glossary of network topology terms). We acknowledge that our study did not capture simultaneous co-locations between individuals but, given that we tracked individuals for multiple days, we consider *N_ij_* to be a likely realization of all possible connections that can occur between them. A natural derivation of *N_ij_* consists in the generation of affiliation networks that link nodes distanced two units from each other [38]: the network connecting participants by the places they have in common (*N_P_*) and the network linking locations by the participants that visit them (*N_L_*) (Table 1). To account for the time-varying properties of human mobility we developed a “dynamic” version of *N_ij_* [*N_ij_(t)*]. We first quantified the visitation to a given place on an hourly basis during a typical week (Monday to Sunday) by calculating the total number of GPS points observed hourly at each place. If this value exceeded 5 points per hour (equaling 12.5 tracking minutes, as GPS loggers collected data every 2.5 min), we considered the person as having stayed at that place rather than being transiently associated with it (e.g., walked in front of it). The visitation data was then used to generate 168 *N_ij_* bipartite graphs describing the hourly movements during a typical week (from Monday to Sunday and from 7∶00AM to 11∶00 PM), which were then sequentially merged to derive *N_ij_(t)*. As 15% of participants had missing information for the periods between 5∶00–7∶00 AM and 11∶00–12∶00 PM (due to some units losing power before they were charged by participants), we excluded those times for the calculation of *N_ij_(t)* in order to obtain a complete set of movement trajectories. *N_ij_(t)* differs from *N_ij_* in that edges represent potential co-location for individuals in space and time rather than space only. The affiliation network *N_P_(t)* was estimated as before and, for each time slice, basic topological indices (described in Table 1) were calculated [38].

**Table 1:** Glossary of network topology terms used in the manuscript.

Network attribute	Definition
Node degree of *N_ij_*	Number of visited location per each person (i) or number of visitors at a given location (j).
Node degree of *N_L_*	Number of other places a place is connected to given the mobility of its visitors.
Node degree of *N_P_*	Number of other individuals an individual is connected to given his/her movement into multiple places.
Component of *N_P_*	A fragment of the network (sub-network) involving only individuals interconnected with each other.
Number of components of *N_P_*	This value indicates how many isolated sub-networks are present inside the full network.
Size largest component N_P_	The number of people included in the largest sub-network
Shortest path of *N_P_(t)*	The average number of intermediate people needed to reach two participants taken at random in the network
Shortest path infection network	The average number of infected people needed to track the pathway of infection from two randomly selected individuals in the network.

The hourly regularity of movement (*R(t)*) quantified the degree of recurrence to places identified as highly visited by participants. To calculate *R(t)* we first identified the location each participant spent most of his/her time on an hourly basis (*X_i,d,h_* for participant *i*, day *d*, hour *h*). *X_i,d,h_* represents the place that received the highest number of visits at a given hour of the day (for instance, school X could have been the place most visited by participant A at 11∶00 AM). We used *X_i,d,h_* to calculate a binary variable, *X_ML_*, indicating whether a participant was at his/her most visited location at hourly intervals during the tracked hours of 7∶00 AM to 11∶00 PM (*X_ML_∼bin(1 if participant was at X_ML_; 0 otherwise)*). *R(t)* was calculated as the proportion of all tracked individuals (*N*) that were found at their most visited location (*X_ML_*) as follows: 
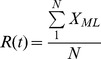
. A high value of *R(t)* indicates that a high proportion of individuals were found at their most visited location. For example, a value of *R(t)* of 0.28 at 1∶00 PM on Wednesday means that 28% of people were visiting their *X_ML_* at that time. Although differently calculated, *R(t)* has the same interpretation as the *R(t)* function derived by Song et al. [26] and used to quantify human mobility from cell-phone data.

#### Modeling disease transmission within an Iquitos neighborhood

As a case study, we theoretically assessed the effect of human mobility within an Iquitos neighborhood on the epidemic propagation of a directly transmitted pathogen by developing a detailed individual-based simulation model (see “Epidemic Model” section in File S1 for a detailed description). The model relied on mobility estimates inferred from the GPS data-loggers to develop a dynamic individual-based bipartite contact network between 1,000 individuals and a hypothetical landscape of 3,900 locations representing 3,000 houses, 630 work-places and 270 commercial areas. This configuration allowed modeling pathogen transmission within a self-contained neighborhood like the ones mobility metrics were obtained from. Contact parameters for each individual (i.e., number of locations visited, time spent at each location, type of location visited) were stochastically derived by randomly sampling from each parameter’s distribution. The model did not account for demographic or social structures (i.e., age, sex or occupation) and was developed using the average population values for each parameter. In order to account for variations in movement and visitation patterns, we simulated contacts at 15-minute intervals for a total of 10,000 time points (equal to 105 days). The model accounted for individual variations in the structure of daily routines: from highly structured routines in which a significant proportion of individuals spent a large proportion of their weekdays on a single location (equivalent to a work-site) to highly unstructured routines in which the duration of visitations was more evenly distributed among all locations. The main parameter defining the degree of structure of an individual’s routine was *μ*, representing the mean value of the exponential distribution of the time spent (in hours) at a given location (section “Epidemic Model” in File S1 and Figure S7 in File S2). The higher the value of *μ*, the more structured a routine (i.e., the more it was focused on a single location).

We modeled the effect of changes in *μ* on the transmission dynamics of a directly transmitted pathogen. A SEIR compartmental model [15] was coupled to the inferred contact network to describe the infection status of each individual. Transmission probabilities and incubation and infectious periods were set at 0.5, 2 days and 4 days, respectively (see Table S5 in File S2 for all the parameter values of the SEIR model); such values are within the ranges observed for directly transmitted infectious diseases such as human influenza virus [39], [40]. The model assumed all infections were symptomatic, and that no behavior change (e.g., locations visited or number of contacts at each location) occurred due to infection. A total of 50 simulations were run for each one of the four *μ* scenarios modeled. The models were initialized by introducing a single infected individual (randomly selected on each simulation) in a fully susceptible population. For each simulation we identified the contact structure emerging from the introduced infection (see videos S1–S4 on File S3) and calculated the epidemic curve and the epidemic’s effective reproductive number (*R_e_*) [41]. We defined *R_e_* as the average number of secondary cases generated by any infectious individual during the duration of the outbreak.

#### Data Limitations

Inherent limitations related to the implementation of GPS technology need to be considered when interpreting data [28]. People can purposely or inadvertently not wear or carry the GPS units, spatial errors can emerge due to poor satellite geometry or multipath signal errors, data collection inside buildings is null or significantly reduced, transiently visited locations can be missed due to the choice of large collection frequencies or due to the monitoring interval [34]. Despite the unprecedented quality of our dataset, we acknowledge that the information derived may underestimate the full repertoire of movements occurring at the spatial and temporal scales considered. Additionally, given our sample size (number of tracked individuals), we did not have enough information to assess temporal variations in routine patterns during and after holidays. Nonetheless, this issue is common to all methods for capturing mobility data at very fine spatial scales. Because GPS units were exchanged twice a week, we asked individuals whether they forgot to use the units at every exchange and found high (>80%) participant compliance. One of the keys for the successful tracking of individuals was the partnership between social, behavioral and spatial scientists who, via focus group discussions, identified and addressed potential concerns people may have regarding the use of GPS units [33].

## Results

### Quantifying Human Mobility Kernels and Spatial Behavior

Human movements outside the city were rare, accounting for only 15.4% of the 2,299,718 GPS locations (Figure 1A). Such low out-of-city movement likely emerged from the high cost associated with long-distance transportation and the geographic isolation of Iquitos. Most (81.0%) movements occurred within 1 km of each individual’s home (Figure 1B), indicating a highly focal kernel of human movement within the city. Indeed, the probability of movement outside an individual’s home *P(Δd)* followed an exponential decay (Figure 1B; Table S2 in File S2). As age increased, the tail of mobility kernels increased (Figure 1B). Most (86.2%) movements by children 7–15 years old occurred within 1 km of their home; such value decreased only to 84.2% for ages 16–25, 77.4% for ages 26–35, 75.8% for ages 36–45, and 80.9% for ages 46 and older (Figure 1B). Movement kernels of males and females did not differ significantly (Wilcoxon Signed Rank Test, U  = −1.33; *P* = 0.183), but males were more likely to move beyond 1 km than females (41.1% vs 34.3%, respectively, Figure 1C). The directionality of movement from each individual’s home was highly anisotropic when all visited locations were considered (due to movement to towns located north and south of the city, Figure 1A and Figure 1D). The spatial anisotropy significantly decreased when only movements within the city were considered, becoming non-significant at ≤5 km from an individual’s home (Figure 1D). This pattern was consistent among all age groups (Figure S2 in File S2) and indicates that within a 5 km radius from a person’s home (the distance up to which most movements occur) human trajectories can be considered omnidirectional.

Overall, participants visited an average ± SD of 5.8±3.6 places over the 2 weeks of monitoring, and the probability distribution of the number of visited places followed a Weibull distribution (Figure 1E and Table S3 in File S2). Most routinely visited places were residential (57.6%), followed by commercial (16.8%), educational (8.5%) and recreational spaces (6.4%) (Figure 1F). The relative frequency of visits to each type of place varied across age groups, with children 7–15 years concentrating most of their trips on residential and educational spaces (53.1% and 14.1%, respectively) and adults on residential and commercial spaces (57.9% and 19.3%, respectively) (Figure S3 in File S2). The duration of visitations to each place was exponentially distributed, with most (78.2%) visits lasting less than 2 hours (Figure S4 in File S2).

**Figure 2:**
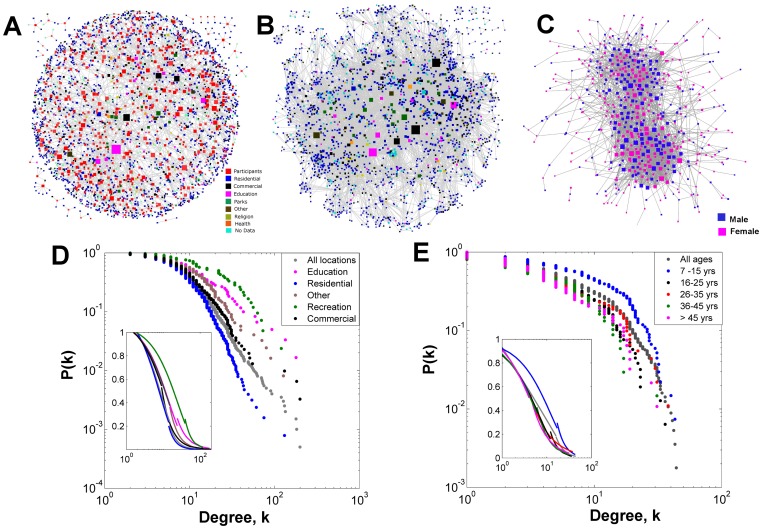
Mobility networks inferred from GPS data-logger data. (A) Networks expressed as an undirected bipartite (*N_ij_*) graph. *N_ij_* was projected into, *N_L_* describing the connections between locations visited by the same individuals (B) and *N_P_* connecting individuals visiting the same locations (C). Frames (D) and (E) show the empirical (main plot) and predicted (inset) degree distributions for *N_L_* and *N_P_*, respectively. See Table 1 for a glossary of network topology terms. The highly focal movement of individuals made it difficult to meaningfully map *N_ij_*_,_ *N_L_* and *N_P_* in geographic space (Figure S5 in File S2).

Despite living in different houses and neighborhoods, the tracked individuals showed a high degree of connectivity, with the largest network component (out of 22 components) accounting for 96.2% of all individuals and 97.8% of all edges (Figure S5 in File S2). *N_ij_* was projected into its constituent affiliation networks: *N_L_* and *N_P_* (Figure 2). The degree distributions of *N_L_* and *N_P_* were best fitted by a truncated power-law distribution of the form:
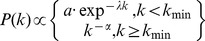
 , where *k_min_* is the distribution’s breakpoint and α and λ scaling parameters (Figure 2D and Figure 2E, Table S4 in File S2).

A deeper analysis of *N_L_* shows that, whereas residential locations were highly visited, the degree of connection between them due to human movement was significantly lower in comparison to non-residential and public spaces (schools and markets) where the daily routines of many individuals converged (Figure 2D). Thus, residential places followed an exponential degree distribution of the form 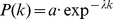
and the remaining location types (excluding recreational spaces, due to low sample size) followed a truncated power-law (Figure 2C and Table S4 in File S2). The *N_P_* network had two components and 20 isolates (Figure 2C). The main component accounted for 96.2% of all individuals, and had a diameter of 11, a density of 0.017 and an average path length of 3.95. The network’s degree distribution was similar for males and females (α = 3.06 and 3.03, respectively and *k_min_*  = 15 and 13, respectively), but differed among age groups (Figure 2E). The low heterogeneity in the number of contacts of children and teenagers is likely the result of the reduced number of public locations they routinely visit (Figure 1E).

#### Dynamic contacts

**Figure 3:**
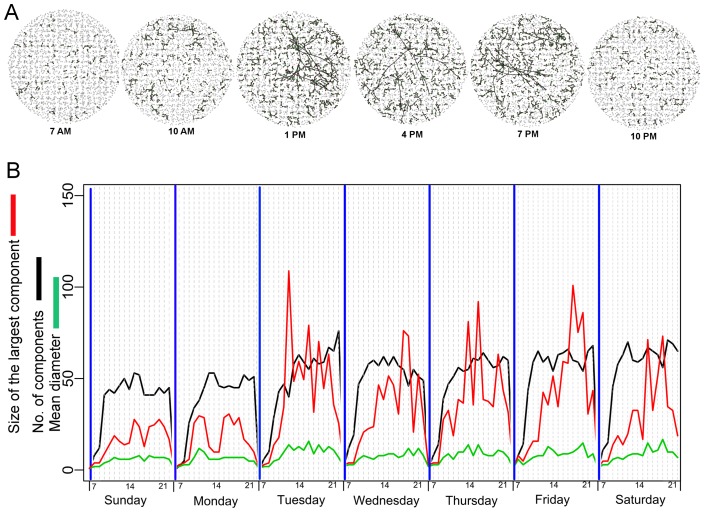
Dynamic mobility networks inferred from GPS data-logger data. (A) Time-varying bipartite graphs (*N_ij_(t)*) showing the variability in the connectivity in space and time among individuals during a typical day (e.g., Tuesday). (B) Time-series of different topological descriptors applied to *N_P_(t)*. As GPS units were turned off from 11∶00 PM to 6AM we excluded this time period from the figure.

**Figure 4:**
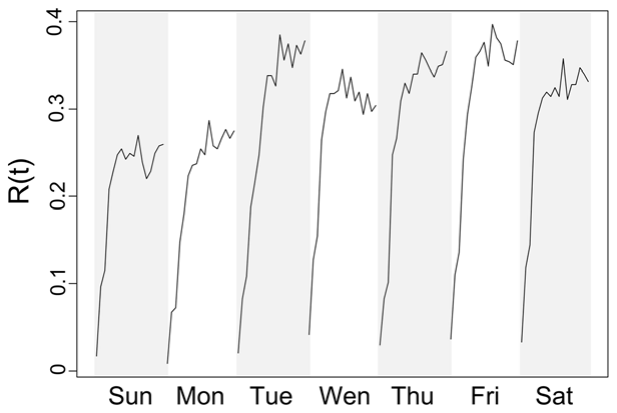
Regularity with which each participant was found at his/her most visited location over a typical week (*R(t)*) from 7∶00AM to 11∶00PM. A high value of *R(t)* indicates that individuals spent a significant proportion of their time at the most visited location (equivalent to work or school, where people could spend between 6 and 8 hours every day).

Figure 3A shows *N_P_(t)* for different time slices of a single day (ranging from early morning to evening) and shows that, as the day progresses, the connectivity between individuals increases, peaking at mid-day and late afternoon and decreasing again in the evening, when people return back home. This time-varying and recurrent pattern of human mobility and co-location can be quantitatively described by three network metrics: the size of the largest component, the number of components and the average shortest path of *N_P_(t)* at every time slice (Figure 3B and Table 1). Whereas the average shortest path of *N_P_(t)* remained fairly constant over time (the mean ± SD over all time slices was 6.5±3.5) the temporal pattern in the size of the largest component and number of components showed significant temporal heterogeneities. Small shortest path values as the ones observed for *N_P_(t)* (ranging between 4 and 9) are characteristic of “small-world” topologies in which very few contacts are needed to reach highly distanced individuals [42], [43]. Individuals moved more after mid-morning and from Tuesday to Saturday, and the overall individual hourly peaks of connectivity (represented by the size of the largest component) did not follow any temporal pattern consistent with repetitive and structured mobility routines (Figure 3B, Figure S6 in File S2). Indeed, only up to 38% of the tracked individuals showed some degree of regularity in their routine (Figure 4), a significant deviation from the ∼60% regularity reported for the hours of 7∶00 AM to 11∶00 PM for people living in an industrialized nation and derived from cell-phone records [Figure 3A from Song et al. [26]]. *R(t)* showed its lowest value at 7∶00 AM, increasing exponentially thereafter (Figure 4), as observed for *N_ij_(t)* and *N_P_*. These estimates emphasize the “small world” structure embedded in daily human mobility networks, and the emergence of temporally unstructured co-locations consistent with the occurrence of highly informal daily mobility routines.

#### Modeling disease transmission within an Iquitos neighborhood

**Figure 5:**
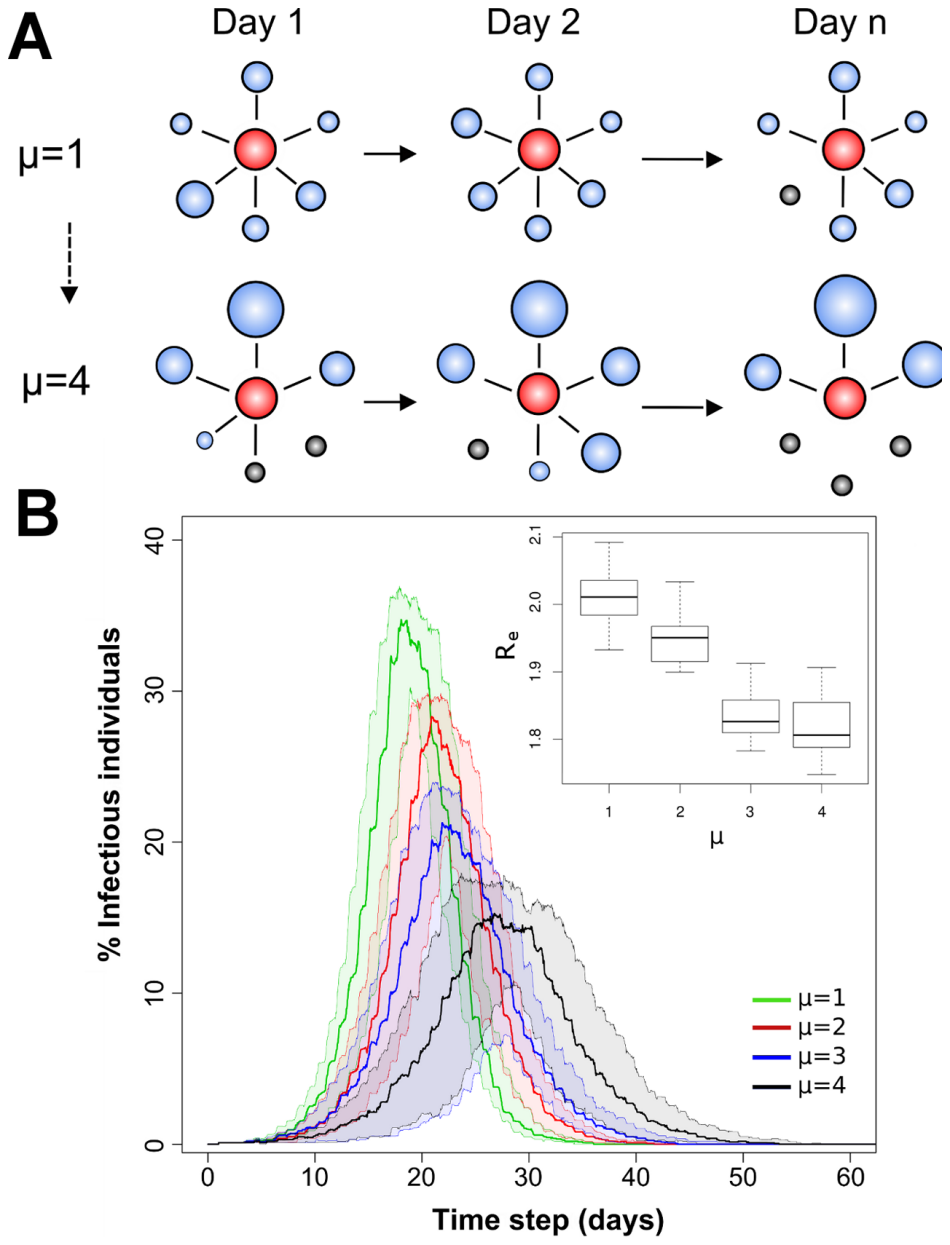
Impact of variable mobility routines on infectious disease dynamics. (A) Diagram outlining the parameter µ from the individual-based model. Red bubbles indicate individuals whereas grey bubbles the locations that belong to their mobility routine. The larger the size of a bubble the more time an individual spent on a location. Some locations may not be visited every day. Individuals who visit and allocate their time into multiple locations have an unstructured routine (*μ*  = 1) whereas individuals who every day spend a significant proportion of their time at one or very few locations have structured routines (*μ* = 4). (B) Epidemic curve and mean reproductive number (*R_e_*) for various scenarios simulating the transmission of an influenza-like pathogen within an Iquitos neighborhood. Models accounted for different levels of structure on an individual’s daily routine (from *μ = *1 to *μ = *4). Confidence bounds were calculated from 50 simulations performed under each scenario.

To assess the implications of highly local and heterogeneous movements on disease dynamics we developed a dynamic contact network individual-based simulation model quantifying the transmission and propagation of a directly transmitted pathogen within an Iquitos neighborhood (Figure 5A and “Epidemic Model” section in File S1). To capture the occurrence of unstructured mobility routines as the ones quantified from our data, we used the parameter *μ*, representing the mean time spent visiting a given location (Figure 5A and Figure S8 in File S2). When routines were highly structured (*μ* = 4) individuals spent a significant proportion of their time on the same location (equivalent of home or work) and their contact networks were highly dispersed in comparison with individuals with highly unstructured routines (*μ* = 1) for which more infectious contacts were identified (Figure S8 in File S2 and Videos S1–S4 on File S3). The contact network derived under a scenario in which *μ* = 1 included one component encompassing 95.1% [range across simulations, 92.0–96.0%] of all infected individuals (Figure 5 and Table 2). The percentage of infections decreased to 88.4% [87.2–90.5%] for a scenario with *μ* = 2, 87.9% [86.1–88.5%] for *μ* = 3 and 79.4% [76.2–80.5%] for *μ* = 4 (Figure 5 and Table 2). With decreasing values of µ, the epidemic’s final size and mean reproductive number, *R_e_*, all increased (Figure 5 and Table 2). Epidemics simulated under a highly unstructured mobility routine (*μ* = 1) produced 20% more cases than epidemics simulated under a structured routine (*μ* = 4) (Figure 5, Table 2 and Figure S8 in File S2, videos S1–S4 on File S3), emphasizing the dramatic impact that dynamic and heterogeneous contacts have on the spatial and temporal dimensions of infectious disease transmission.

**Table 2:** Topological metrics of the transmission networks (median, Q1-Q3) obtained after running 50 simulations of the individual based model of the transmission of a directly transmitted pathogen during a 105 day period.

Scenario	Duration of epidemic (days)	No. infected individuals	No. locations with infections	No. infectious contacts	Contact network shortest path
Μ = 1	45 (43–48)	951 (920–960)	339 (333–350)	1680 (1589–1732)	16 (15–17)
Μ = 2	51 (48–56)	884 (872–905)	347 (339–351)	1629 (1561–1660)	18 (16–18)
Μ = 3	58 (57–62)	879 (861–885)	331 (317–340)	1472 (1391–1496)	18 (17–18)
Μ = 4	70 (65–75)	794 (762–805)	325 (317–330)	1138 (1268–1440)	19 (18–20)

µ represents the degree of structure on an individual’s routine (from highly unstructured, µ = 1, to highly structured, µ = 4). See Methods and File S1 for details.

## Discussion

Global urban development is dominated by significant inequalities between the developed and developing world [31]. Whereas developed cities offer important opportunities for economic and social development and act as focal points for economic growth, innovation, and employment, rapid urban growth throughout the developing world is seriously impacting the capacity of most cities to provide adequate services for their citizens (particularly the urban poor), challenging the notion of sustainable urban development in resource-poor settings [31], [44], [45]. As location-aware technologies become more pervasive and affordable, a unique opportunity for quantitatively characterizing complex social systems in developing countries is now emerging. Uncovering the basic mechanisms governing complex human behaviors in resource-poor urban environments is paramount for developing better infrastructure, fostering local economic development and responding to the emergence, transmission and propagation of infectious disease threats.

Long-term spatial and temporal scaling patterns of human movement have been mathematically described most frequently by power-law distributions emerging as a consequence of the wide availability of communication, infrastructure and transportation options associated with developed urban centers [18], [24]–[26]. An earlier study using cell phone records from over 3.5 million customers living in 8 Chinese cities described human mobility as highly local (more than 90% of movements occurred within 5 km from each other) and proportional to the geographic extent and compactness of the urbanized area [46]. Similarly, Iquitos residents spent most of their time within the bounds of the city, engaging in social, commercial or recreational activities in close proximity (∼1km) to their home. Given that most people in Iquitos lack personal means of transportation, movements within and outside the city are significantly affected by economic and time constraints. Furthermore, there was no significant difference in the movement kernels associated with each type of place (e.g., residential, commercial, recreational), emphasizing the importance of distance, rather than type of activity, in modulating human mobility within this tropical urban environment. Our results, using a more spatially-defined data source, are in agreement with previous findings [46] and suggest that power laws may not the most important component of human trajectories within resource-poor environments. Rather, space imposes a significant friction to human movement, giving rise to highly local within-city mobility kernels, better described by exponential functions.

Human activities are a function of preferences, tastes, obligations, information, habits and financial circumstances [12]. In developed urban societies, activities to which an individual commits significant time/effort constrain the ordering of other routine activities, giving rise to highly predictable human trajectories [13], [24], [26]. Mobility patterns in such environments are generally bound by a journey-to-work structure in which accessibility and economic/social opportunities determine the occurrence of regular and recursive mobility patterns [20], [24], [26]. With the exception of children and adolescents up to 25 year of age, who spent significant time at schools/colleges, most of the working-age individuals in this study lacked a repetitive pattern of visitation to specific locations that is compatible with a journey-to-work structure. Instead, they engaged in various activities during a regular day, visiting an average [range] of 6 [1]–[21] places, and potentially interacting with other individuals visiting or residing in them. Such temporally unstructured routines affected the daily and hourly connectivity and architecture of the inferred mobility networks, and played a major role on the persistence and extent of the modeled epidemics.

Recent infectious disease modeling efforts have aimed at incorporating realism by considering human commuting behaviors within large-scale meta-population type modeling frameworks [11], [20], [22], [23]. Mobility quantifications obtained from industrialized countries outline the importance of commuting between census areas, which can be up to an order of magnitude higher than non-commuting movements [8], [22]. When regular movements (emerging from the commuting behavior of workers) between population areas are accounted for, the epidemic speed and invasion threshold of pathogen transmission are significantly lengthened [11], [20]. These findings, obtained from various data sources and modeling frameworks, outline the importance of human behavior in disease dynamics and challenge the utility of models excluding heterogeneous contact patterns as predictive tools for public health response. Our study extends previous work by: a) analyzing data at much finer spatial and temporal scales and level of resolution (i.e., individual movements rather than aggregate movements between districts or cities); b) incorporating a richer repertoire of human mobility behaviors (e.g., number of locations and type of location); and 3) relying on parameter values derived from a resource-poor urban environment. Our simulation models show that human mobility and contacts within Iquitos are highly heterogeneous within a typical week and that, by not accounting for such dynamic contacts, estimates of individual connectivity and transmission of a directly transmitted pathogen could be significantly underestimated.

Results from a series of detailed stochastic simulation models suggest that, in industrialized countries, halting human contact networks by closing highly visited places or by socially distancing individuals could substantially lower pandemic influenza attack rates before a vaccine becomes available, with timely initiation of measures and school closure playing important roles in dampening transmission dynamics [39], [47]. The extent to which such measures may prove effective in resource-poor cities such as Iquitos remains poorly understood [48]. The high variability in observed mobility routines may make it difficult to enforce household isolation or to identify which premises (work-places or public spaces) will need to be closed, because individuals spend a significant amount of time at multiple locales. Furthermore, given the highly variable movement patterns observed among children, home isolation after school closures may be difficult to enforce. It is estimated that most (>90%) of the mortality exerted by a potential pandemic influenza epidemic would occur in developing countries [49], where vaccine and antiviral stockpiles are minimal [48]. The lack of detailed mathematical models parameterized to estimate infectious disease transmission dynamics in such settings limits local and regional public health offices’ ability to enforce containment measures or plan emergency preparedness strategies that are context specific [48]. Our findings reveal inherent complexities characterizing human mobility with implications for the understanding and contextualization of infectious disease dynamics in developing countries. Results from our study contribute new empiric information for the development of more realistic infectious disease models that account for the complex, fine-grained and dynamic nature of human interactions that characterize resource-poor urban environments.

## Supporting Information

File S1 **Supplementary Methods.**
(DOCX)

File S2 **Supplementary Figures and Tables.**
(DOCX)

File S3 **Supplementary Videos.** Dynamic representation of the pathway followed by the introduced infection through the modeled population. Each video represent the temporal sequence of the infection when μ = 1 (Video S1), μ = 2 (Video S2), μ = 3 (Video S3) and μ = 4 (Video S4).
(ZIP)
