# Supplementary material for: Using GPS Technology to Quantify Human Mobility, Dynamic Contacts and Infectious Disease Dynamics in a Resource-Poor Urban Environment
Source: PLoS One. 2013 Apr 8;8(4):e58802. doi: 10.1371/journal.pone.0058802 (PMC3620113; doi:10.1371/journal.pone.0058802)
Supplement: File S1 — Supplementary Methods. (DOCX) [file pone.0058802.s001.docx]

***File S1***

**Using GPS technology to quantify human mobility, dynamic contacts and infectious disease dynamics within a resource-poor urban environment**

Gonzalo M. Vazquez-Prokopec^a,1,2^, Donal Bisanzio^1^, Steven T. Stoddard^2,3^, Valerie Paz-Soldan^4^, Amy C. Morrison^3^, John P. Elder^5^, Jhon Ramirez-Paredes^6^, Eric S. Halsey^6^, Tadeusz J. Kochel^7^, Thomas W. Scott^2,3^, Uriel Kitron^1,2^

*^1^Department of Environmental Studies, Emory University, Atlanta, GA, USA; ^2^ Fogarty International Center, National Institutes of Health, Bethesda, MD, USA*; ^3^*Department of Entomology, University of California, Davis, CA, USA; ^4^Tulane University School of Public Health and Tropical Medicine, New Orleans, LA, USA; ^5^ Graduate School of Public Health, San Diego State University, San Diego, CA, USA; ^6^U.S. Naval Medical Research Unit, Lima and Iquitos, Peru.* ^7^Naval Medical Research Center, Silver Spring, MD;

**^a^Corresponding author:** [gmvazqu@emory.edu](mailto:gmvazqu@emory.edu)

**Supporting Online Material**

Below we provide all relevant details on data collection and analysis, including an explanation of participant recruitment and composition, data collection protocols and a summary of statistical analyses and relevant additional results.

**Content**

**GPS Selection ………………………………………………………………… page 2**

**Subject pool…………………………………………………………………… page 2**

**Data Processing………………………………………………………………. page 3**

**Data Analysis…………………………………………………………………. page 3**

**Epidemic model………………………………………………………………. page 5**

**Supplementary References…………………………………………………… page 9**

**GPS selection.** A series of 15 focus group discussions (totalling 144 adult participants) were conducted in January 2008 to examine whether it would be acceptable for Iquitos residents to wear GPS data-loggers, and to assess the concerns potential participants would have regarding GPS use [[1](#_ENREF_1)]. Based on the focus groups’ results and the technological needs for tracking human movements [[2](#_ENREF_2)], we determined the set of requirements a GPS data-logger unit should meet to track our study participants: a) data storage capacity and battery life capable of recording at least 3 days of data (to ease mass deployment of units and long-term tracking of individuals); b) durable, water resistant and tamper-proof; c) light weight (i.e., < 100 g); d) unobtrusive design (so that it would not be noticed by others, reducing the chances of potential harm from people who might want to steal the units); e) carrying mechanism widely accepted by participants of different age/gender; f) little to no maintenance required by study participants; and g) low cost. From a total of four commercially available and two custom-made GPS data-loggers considered, only one (“Igot-U GT120” Mobile Action Technology Inc.) met all of the above criteria [[3](#_ENREF_3)] and was used in this study.

**Subject pool.** Recruitment of participants was conducted in two separate neighbourhoods of Iquitos, Tupac Amaru and Maynas (Figure S1 in File S2), and aimed to obtain a balanced sample across age groups (7-15, 16-25, 26-35, 36-45 and >45 years of age) and genders (Table S1 in File S2). On-going epidemiological research in the area [[4](#_ENREF_4)] facilitated logistics in GPS deployment and retrieval and was the main determinant in the choice of the study neighbourhoods. Recruitment was performed by trained local technicians, who provided a description of the study together with a pamphlet with specific information about the GPS units and the study in general [[1](#_ENREF_1)]. After providing informed consent each participant was asked to wear a GPS unit on a neck strap at all times. The research team scheduled an exchange of the GPS units every three days to download data, verify function, and recharge batteries. At the time of GPS unit exchange, participants were asked about their experiences with the GPS, whether they had used it, if it had been forgotten and, if so, on what days. A description of the demographics of the subject pool can be found in Table S1 in File S2. Children younger than 7 years were excluded from this study due to the difficulty of having them wear the loggers at all times (and the subsequent increase in positional bias and error in identifying out-of-home locations visited). Tracking was not simultaneous for all participants, and spanned from October 2009 to May 2011, with most (67%) participants being tracked during the year 2010.

**Data Processing.** Data collected by GPS units consisted of a sequence of geographic locations $\left( x_{i},y_{i} \right)$ at time points $t_{i}=0,1,2,\ldots,n$; where $x_{i}= x\left( t_{i} \right)$ and $y_{i}= y\left( t_{i} \right)$. Geographic coordinates $\left( x_{i},y_{i} \right)$ were expressed in meters (Universal Transverse Mercator, Zone 18S; WGS1984 Datum), allowing calculation of the Euclidean distance from each GPS-tagged geographic location to each participant’s home ($x_{home})$, $\Delta d=\sqrt{\left( x_{i}-x_{home} \right)^{2}+\left( y_{i}-y_{home} \right)^{2}}$, and the angle of movement to each location, $\theta=\arctan\left( \frac{y_{i}-y_{\mathrm{home}}}{x_{i}-x_{\mathrm{home}}} \right).$ The relative position of each location with respect to a participant’s house was obtained by calculating a new set of coordinates $\Delta X=\left( x_{i}-x_{home} \right)$ and $\Delta Y=\left( y_{i}-y_{home} \right)$ and plotting them in Euclidean space (Figure S2 in File S2). We applied a velocity filter to eliminate outlier positions (emerging from multipath and other GPS signal errors) by calculating time and distance between successive locations and eliminating those locations over a pre-defined (160 km/h) velocity threshold [[3](#_ENREF_3)].

**Data Analysis.** Maximum Likelihood techniques were applied to fit various statistical distributions (exponential, Weibull, power-law, truncated power-law) to *P(Δd)* (the cumulative probability distribution of the distance of a given GPS location to an individual’s home), the number of places visited, and the degree distribution of *N_P_*, and *N_L_*. We applied a Kolgomorov-Smirnov statistic to determine if the parameters fitted by a power-law distribution were significantly different from such distribution following the methods described in [[7](#_ENREF_7)]. Fits were performed on data for all individuals, for each sex and for each age category. We applied temporal wavelet functions to assess the temporal correlations in the size of the largest component of *N_P_(t)*. We calculated the local wavelet power spectrum by first temporally de-trending the data by using the mean of the series as the fitting function. We then applied a Morlet wavelet structure [[9](#_ENREF_9), [10](#_ENREF_10)] to the de-trended data to generate the wavelet power spectrum (Figure S6 in File S2). The presence of statistically significant (*P*<0.05) periods in the wavelet power spectrum is indicative of the presence of recurrent patterns in the connectivity of individuals, which can be interpreted as the occurrence of temporally structured mobility networks.

**Epidemic model.** As a case study, we quantified the effect of variable daily routines in the epidemic propagation of a directly transmitted pathogen within an Iquitos neighbourhood by generating a stochastic individual-based simulation model. We modelled dynamic contacts between 1,000 individuals and ~4,000 locations (the ratio of people to locations observed in Iquitos) by generating, at 15 minute intervals, bipartite graphs representing the contact between individuals due to their mobility patterns. After the initial conditions of the model were established (see below and Table S5 in File S2) the dynamic contact network was developed following an iterative process of edge construction and destruction based on the combination of permanence time at a location, number of locations belonging to an individual’s routine and time taken to move between locations. The steps followed to develop the dynamic contact networks are described below (the full code for the model is available upon request to the corresponding author):

**Model setup:**

- Create locations:
  - Number of places = 3,990
  - Type of Place. Home, work places, and commercial areas on a 1.0 : 0.21 : 0.09 ratio, respectively (based on estimates from the Iquitos GIS).
  - Set a location’s attractiveness. This represents the preferential attachment of locations to each individual in the network, and allows the generation of the “small-world” structure observed in the data. We used a Power Law Distribution with coefficient λ= 2.8 to generate the probability of a place to be visited by *N* individuals.
  - Operation hours. Each place had different hours in which was open for visits. Houses were open 24hs, work places 12hs and commercial places 16hs.
- Create individuals:
  - Number = 1,000
  - Movement routine. Maximum number of places that belong to an individual’s routine. Based on GPS data. Weibull Distribution with scale= 6.5, shape=1.7.
  - Infection status. All individuals were considered Susceptible at the beginning of the simulation.
  - Assign home location. Allocate *n* people to each “home” location following a Poisson Distribution with mean=6 (based on mean household size in the study neighbourhoods).
  - Assign the possible visited locations to each individual. Each individual was assigned n-1 locations to its routine. Locations with high attractiveness were preferred over locations with low attractiveness.

**Model simulation:**

- Set time step = 15 minutes
- At every time step:
  - Calculate number of open places, given the operation hours and the time of the day.
  - Randomly assign one place to each individual by sampling from the list of potential places belonging to their routine.
  - Set time spent at the location. Randomly sample from an exponential distribution with mean (μ) parameter, μ=1, μ=2, μ=3, or μ=4 (each μ represents a simulated scenario).
  - Update the individual infection status based on infection probabilities and incubation periods (Table S5 in File S2).
  - Calculate how many individuals are Susceptible, Exposed (incubating), Infectious and Recovered at every place.

The parameter of interest in our model was μ, the duration of visitation of a routinely visited location. μ was used as a routine generator, since it allowed varying the degree of structure in an individual’s mobility schedule. Highly structured routines were assumed to follow a pattern in which one location accounts for most of the time spent out of home during a typical week (compatible with work or school). Individuals under an unstructured routine divided their time out of home within multiple locations, with no location playing a role as a major destination. By randomly sampling from a family of negative exponential curves with different mean (Figure S7 in File S2) we generated a gradient of mobility routines: from structured (µ = 4) to highly unstructured (µ = 1). Each curve was truncated at 24 hours, representing the maximum time an individual could spend at a place on a given day. The time visitation data derived from the GPS tracked individuals was best fit by an exponential function with µ = 2.7 (Figure S7 in File S2). Our model also accounted for the time required to travel between locations by randomly assigning a delay in arriving to the next place. The delay was assigned by randomly sampling from a uniform distribution ranging from 15 minutes to 2 hours (with a step of 15 minutes). We considered 2 hrs as the maximum time delay for moving between places.

We coupled the bipartite contact network with a SEIR model for a directly transmitted (i.e., influenza-like) pathogen to quantify the epidemic spread of an infection through a fully susceptible population. The main goal was to assess the impact that empirically-derived temporally unstructured routines may have on infectious disease dynamics. Transmission parameters (summarized on Table S5 in File S2) were derived from known models describing the transmission of a directly transmitted pathogen such as influenza virus [[11](#_ENREF_11), [12](#_ENREF_12)]. Every time step, the model calculated the number of susceptible, exposed (incubating), infectious and recovered individuals. Each individual was tracked, hence individual infection status was continuously updated after the incubation or recovery periods were reached (2 and 4 days, respectively, see Table S5 in File S2). We ran 50 simulations for each level of *μ*.

The model had the following assumptions: a) only one individual can introduce the disease to the system (the model randomly selected an individual as the index case on every simulation); b) individuals do not change their routine during the simulation period; c) individuals return to their “home” and spend the night there (they spend home from 11:00 pm to 6:00 am); c) the modelled population is not demographically structured (i.e, no sex or age differences); d) co-located individuals (those who are at the same place at the same time) have an equal probability of contacting each other; e) after infection, all individuals recover and become immune (no infection-mediated mortality); f) the total population remains constant (1,000 individuals). The model was developed in the NetLogo 4.3 modelling platform (http://ccl.northwestern.edu/netlogo/) and is available upon request to the corresponding author.

We quantified the epidemic spread in the modelled population by calculating the effective reproductive number (*R_e_*) [[13](#_ENREF_13)]. We define *R_e_* as the average number of secondary cases generated by any infectious individual during the outbreak. We were able to calculate *R_e_* directly from the output by looking how many new infections were generated by an individual during his/her infectious period. The value of *R_e_* is generally smaller than the basic reproductive number, *R_0_*, due to the reduction of susceptible individuals through the course of the epidemic [[13](#_ENREF_13)]. We used the output of the model to derive the transmission network representing the propagation of infection across the modelled population (Figure S8 in File S2). The structure of this network only considered those contacts responsible for pathogen transmission. The resulting networks were visually represented using a direct graph in which the position of each node was estimated applying a Kamada-Kawai layout algorithm (Figure S8 in File S2). This visualization method allowed us to underline the hierarchical structure of the network: the center of the graph indicates the first case whereas the periphery of the graph the cases that occurred later in time (Figure S8 in File S2). Using the same methodology we created daily animations of representing the propagation of the pathogen through the network (Videos S1-S4).

**Supplementary References**

1. Paz-Soldan V., Stoddaard S., Vazquez-Prokopec G., Morrison A., Elder J., Kitron U., Kochel T., Scott T.W. 2010 Assessing and maximizing the acceptability of GPS device use for studying the role of human movement in dengue virus transmission in Iquitos, Peru. *Am J Trop Med Hyg* **82**(4), 723-730.

2. Elgethun K., Fenske R.A., Yost M.G., Palcisko G.J. 2003 Time-location analysis for exposure assessment studies of children using a novel global positioning system instrument. *Environ Health Perspect* **111**(1), 115-122.

3. Vazquez-Prokopec G.M., Stoddard S.T., Paz-Soldan V., Morrison A.C., Elder J.P., Kochel T.J., Scott T.W., Kitron U. 2009 Usefulness of commercially available GPS data-loggers for tracking human movement and exposure to dengue virus. *Int J Health Geogr* **8**, 68. (doi:1476-072X-8-68 [pii]

10.1186/1476-072X-8-68).

4. Morrison A.C., Minnick S.L., Rocha C., Forshey B.M., Stoddard S.T., Getis A., Focks D.A., Russell K.L., Olson J.G., Blair P.J., et al. 2010 Epidemiology of dengue virus in Iquitos, Peru 1999 to 2005: interepidemic and epidemic patterns of transmission. *PLoS Negl Trop Dis* **4**(5), e670. (doi:10.1371/journal.pntd.0000670).

5. Hu D., Wang C.-L. 2007 GPS-Based Location Extraction and Presence Management for Mobile Instant Messenger. In *Embedded and Ubiquitous Computing* (pp. 309-320.

6. Morrison A.C., Gray K., Getis A., Astete H., Sihuincha M., Focks D., Watts D., Stancil J.D., Olson J.G., Blair P., et al. 2004 Temporal and geographic patterns of *Aedes aegypti* (Diptera: Culicidae) production in Iquitos, Peru. *J Med Entomol* **41**(6), 1123-1142.

7. Clauset A., Shalizi-Rohilla C., Newman M.E.J. 2009 Power-law distributions in empirical data. *SIAM review* **51**, 661-703.

8. Rosenberg M., Kenkel N. 2004 Wavelet analysis for detecting anisotropy in point patterns. *Journal of Vegetation Science* **15**(2), 277-284. (doi:10.1658/1100-9233(2004)015[0277:wafdai]2.0.co;2).

9. Shumway R.H., Stoffer D.S. 2006 *Time series analysis and its applications: with R examples*. 2nd [updated] ed. New York, Springer; xiii, 575 p. p.

10. Cazelles B., Chavez M., Magny G.C.d., Guégan J.-F., Hales S. 2007 Time-dependent spectral analysis of epidemiological time-series with wavelets. *Journal of The Royal Society Interface* **4**(15), 625-636. (doi:10.1098/rsif.2007.0212).

11. Germann T.C., Kadau K., Longini I.M., Jr., Macken C.A. 2006 Mitigation strategies for pandemic influenza in the United States. *Proc Natl Acad Sci U S A* **103**(15), 5935-5940. (doi:10.1073/pnas.0601266103).

12. Halloran M.E., Ferguson N.M., Eubank S., Longini I.M., Cummings D.A.T., Lewis B., Xu S., Fraser C., Vullikanti A., Germann T.C., et al. 2008 Modeling targeted layered containment of an influenza pandemic in the United States. *Proceedings of the National Academy of Sciences* **105**(12), 4639-4644. (doi:10.1073/pnas.0706849105).

13. Wallinga J., Teunis P. 2004 Different Epidemic Curves for Severe Acute Respiratory Syndrome Reveal Similar Impacts of Control Measures. *Am J Epidemiol* **160**(6), 509-516. (doi:10.1093/aje/kwh255).
