# Supplementary material for: Using GPS Technology to Quantify Human Mobility, Dynamic Contacts and Infectious Disease Dynamics in a Resource-Poor Urban Environment
Source: PLoS One. 2013 Apr 8;8(4):e58802. doi: 10.1371/journal.pone.0058802 (PMC3620113; doi:10.1371/journal.pone.0058802)
Supplement: File S2 — Supplementary Figures and Tables. (DOCX) [file pone.0058802.s002.docx]

***File S2***

***Supplementary Figures and Tables***

**Figure. S1.** Base map of Iquitos indicating the location of the study neighborhoods (Maynas in the North and Tupac Amaru in the South). Inset shows the location of Iquitos within Peru.
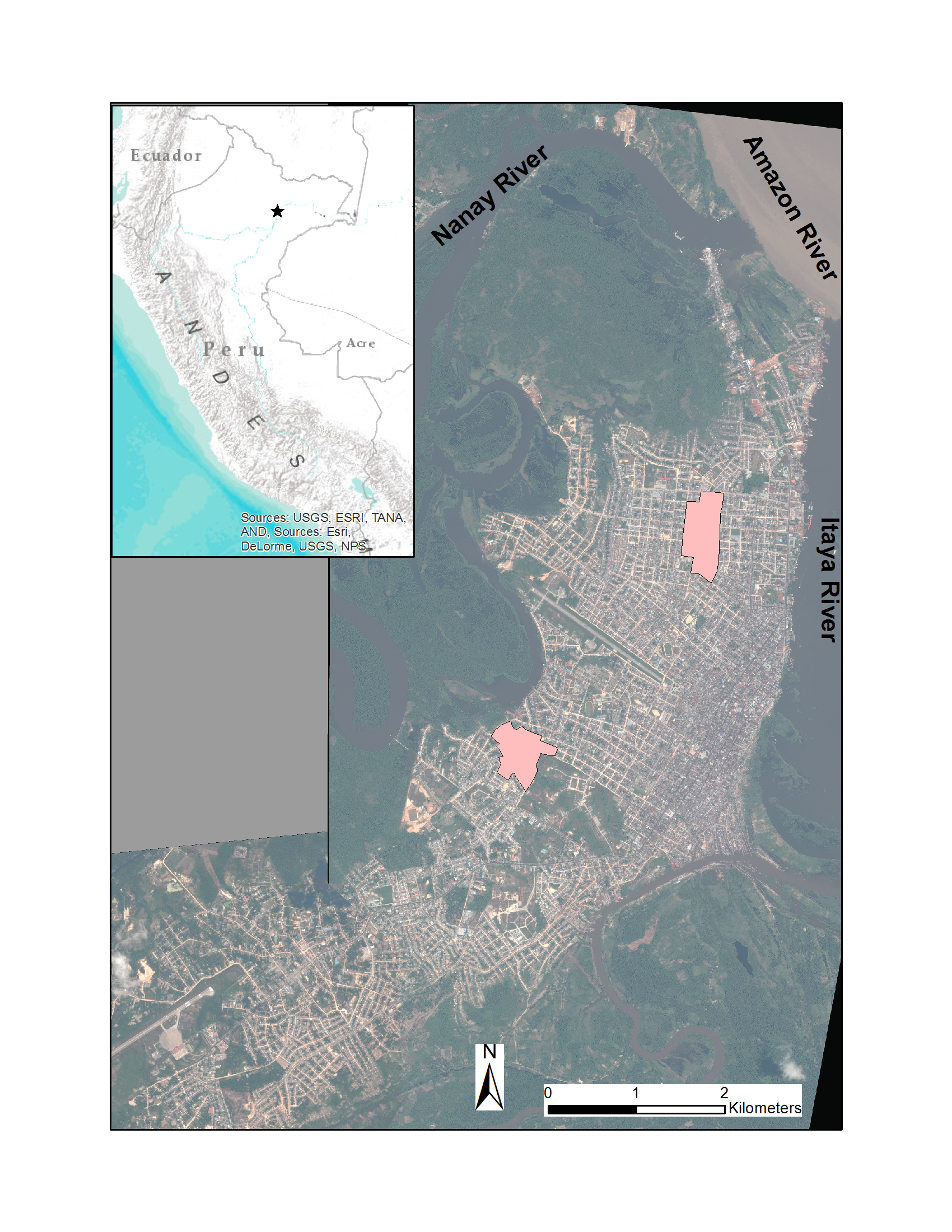


**Figure S2.** Spatial arrangement of GPS locations around the home of the tracked participants (dots) and interpolated density of points at 10 m intervals (surface) for each age group: (A) 7-15 years, (B) 16-25 years, (C) 26-35 years, (D) 36-45 years, (E) older than 46 years. All individuals showed an omnidirectional pattern of movement around the house < 5 km.


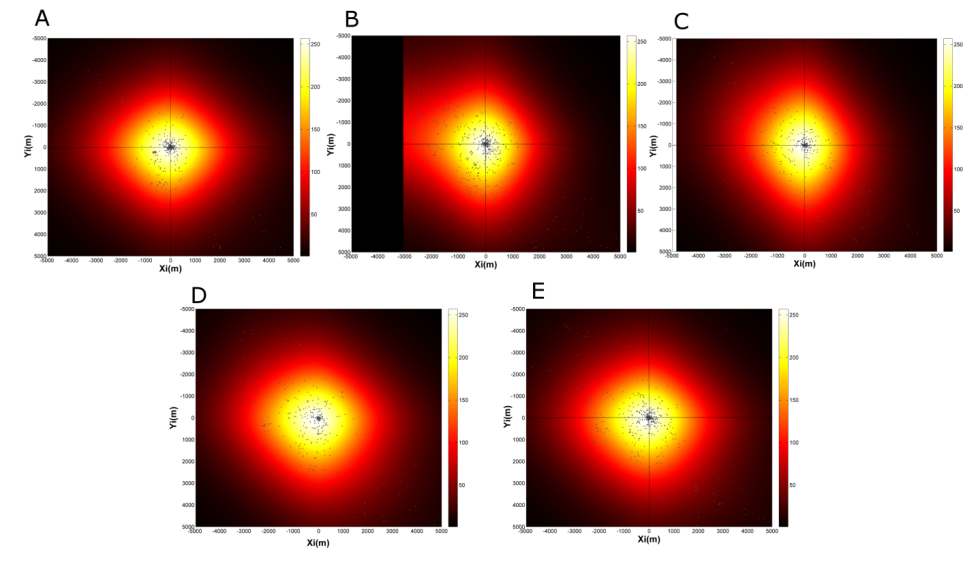


**Figure S3.** Relative frequency of the number of places visited by different age groups and genders, ordered by land-use type.

**
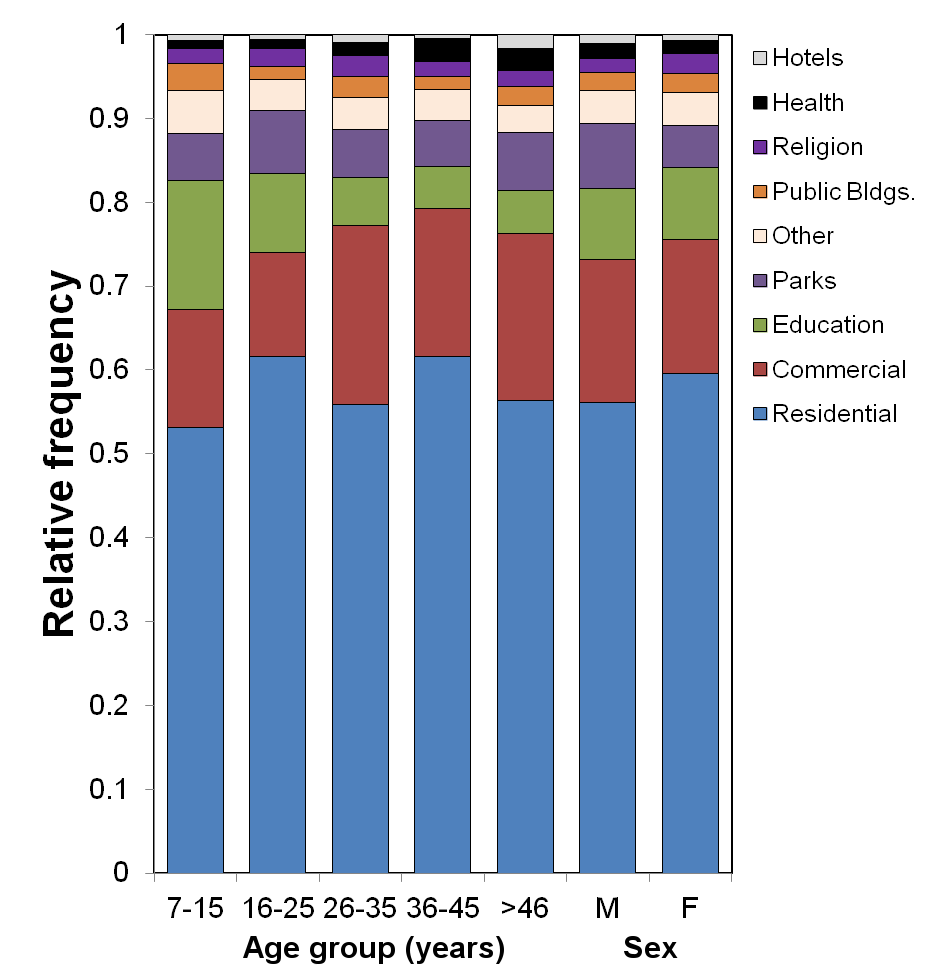
**

**Figure S4.** Frequency distribution of the duration of each visit. The data was best fit by a negative exponential function with coefficient μ = 2.7 [95% CI, 2.5-2.9].

**
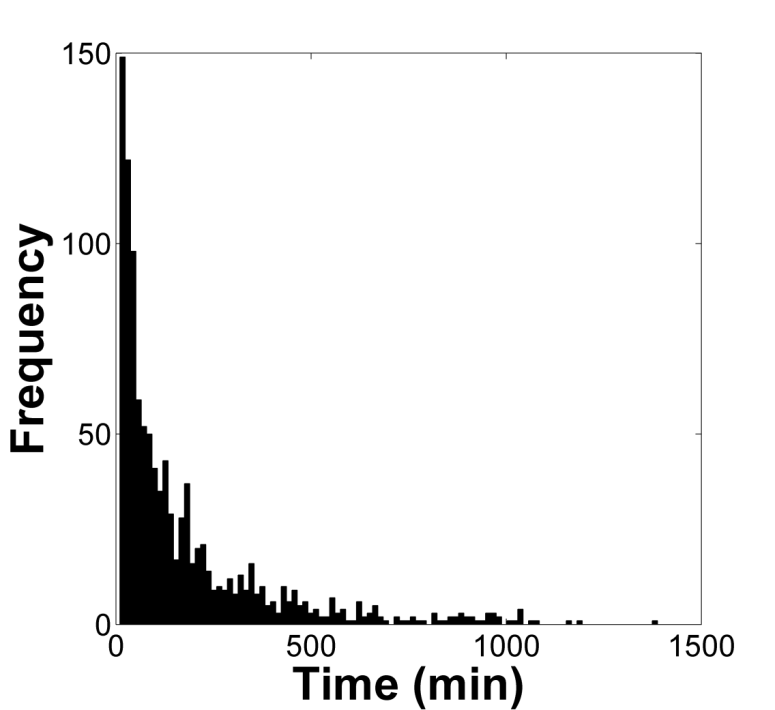
**

**Figure S5**. Spatial representation of the bipartite graph connecting individuals to the places they visited while tracked with a GPS data-logger (*N_ij_*). Black circles indicate individuals in their houses and yellow circles the places visited. Edge colors separate individual participants. Inset shows the a-spatial version of *N_ij_*_,_ represented using a spring-embedded circular layout (red indicates participants and the other colors represent different land-use types). *N_ij_* had a diameter of 12, a path length of 5.45, an average of 2.76 neighbors and a density of 0.001.


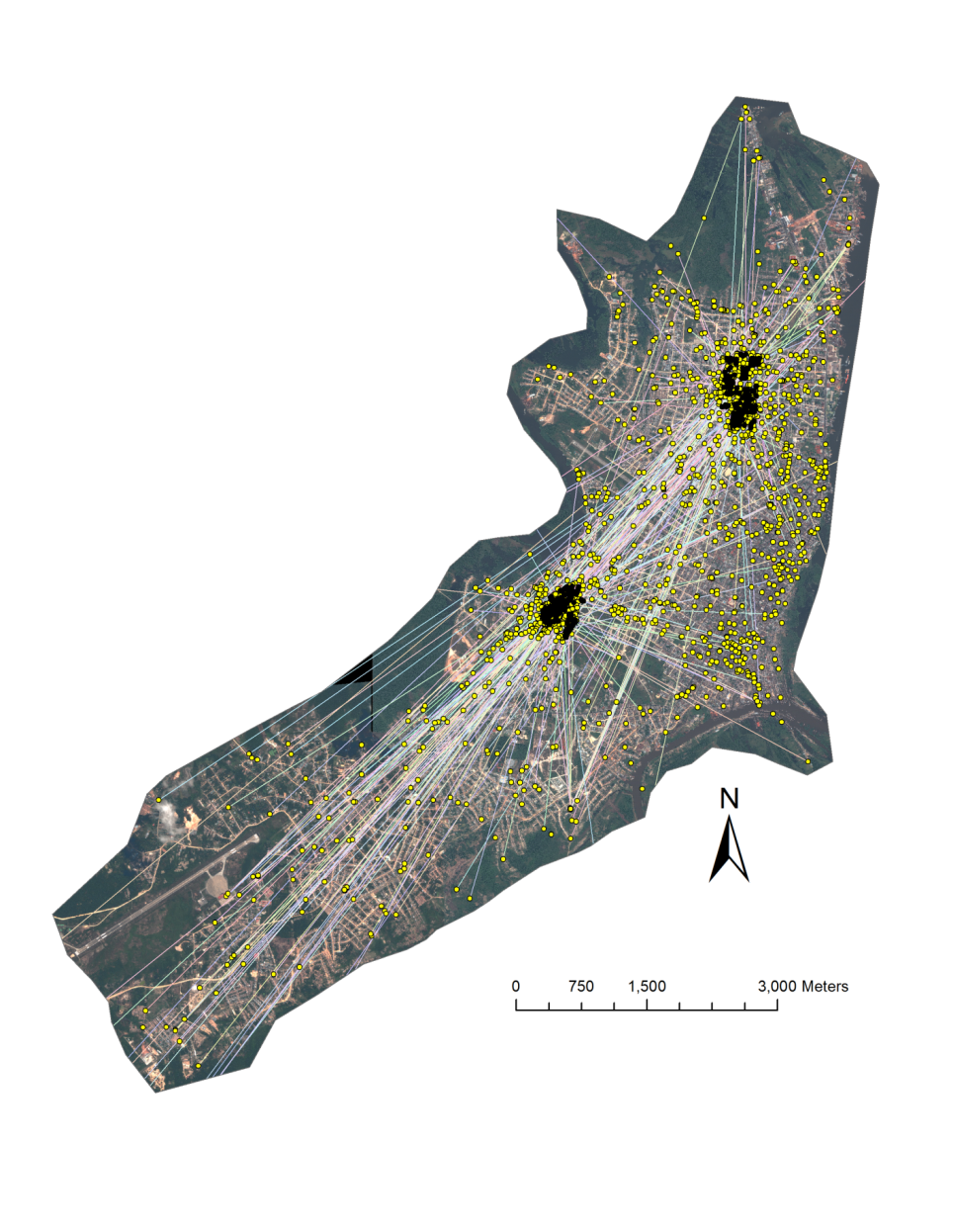


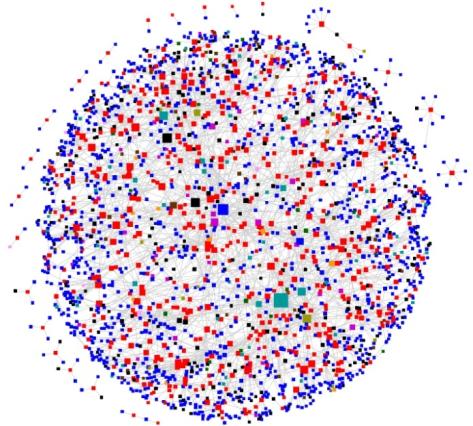


**Figure S6.** Wavelet temporal analysis of the size of the largest component of *N_P_(t)* at 1 hour intervals for a typical week. The top panel shows the size of the largest component after temporal de-trending (i.e., fitting the data using the mean of the series) and the lower panel the wavelet power spectrum. Values outside the striped diagonal lines indicate the cone of influence (the area within which results are not influenced by the edges of the data). The ellipses indicate period (in hours) up to which the cluster size is significant (P < 0.05). Only a slight correlation was observed at a period of 14-16 h between Thursday and Friday, indicating the connectivity structure among individuals was not consistently repeated over a typical week.

**
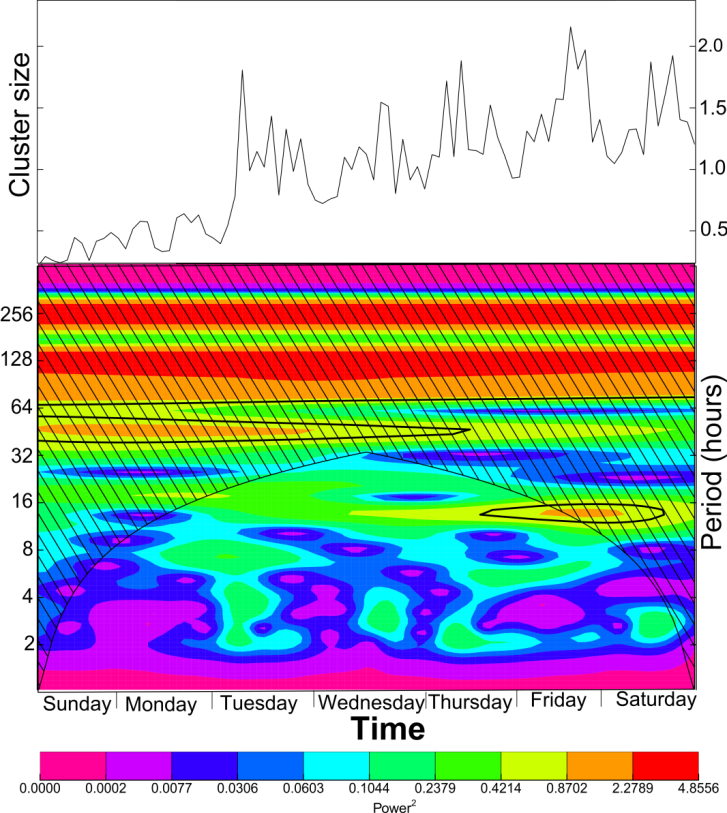
**

**Figure S7.** Density distribution of the number of hours an individual can spend visiting an individual place on a given day. The parameter µ represents the mean value of a negative exponential function. The observed time distribution for the GPS tracked individuals from Iquitos was best fit by an exponential function with µ = 2.7 [95% CI, 2.5-2.9, *P*<0.05].

**
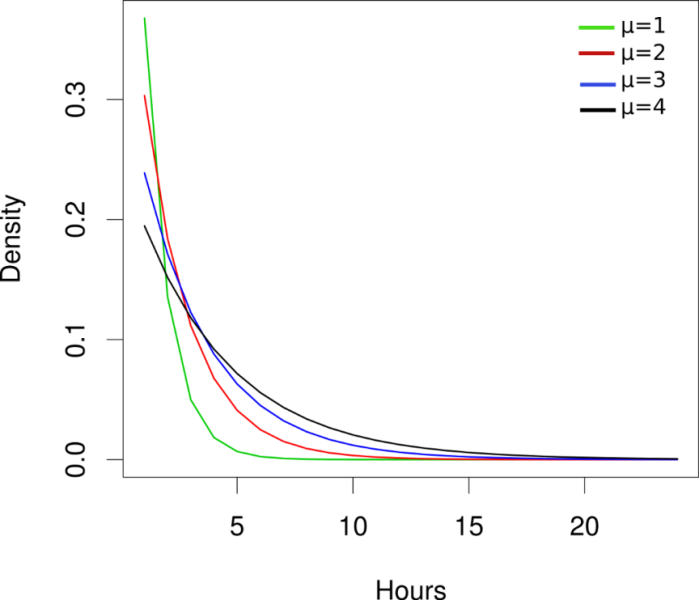
**

**Figure S8.** Representation of the pathway followed by the introduced infection as it propagated through the modeled population. Each panel shows one randomly selected realization (out of 50 performed) for each of the different scenarios (μ = 1 for highly unstructured to μ = 4 for highly structured mobility routines). The center of each graph indicates the epidemic index case. Each graph was drawn using a layout based on the Kamada-Kawai algorithm. The size of each node is proportional to its degree. The graphs not only outline the way the infection propagated, but also the significant heterogeneity across individuals in terms of their contribution to transmission. See Videos S2-S5 for an animated view of the propagation of infection through each network.

**Supplementary Videos**

**Video S1.** Dynamic representation of the pathway followed by the introduced infection through the modeled population. This directed graph shows the temporal sequence of the infection when μ = 1.

**Video S2.** Dynamic representation of the pathway followed by the introduced infection through the modeled population. This directed graph shows the temporal sequence of the infection when μ = 2.

**Video S3.** Dynamic representation of the pathway followed by the introduced infection through the modeled population. This directed graph shows the temporal sequence of the infection when μ = 3.

**Video S4.** Dynamic representation of the pathway followed by the introduced infection through the modeled population. This directed graph shows the temporal sequence of the infection when μ = 4.

**Table S1.** Demographics of study participants enrolled in GPS tracking study in the city of Iquitos, Peru.

|  |  |  |  | No. of participants by age group | | | | |
| --- | --- | --- | --- | --- | --- | --- | --- | --- |
| Neighborhood | No. participants | No male | No. female | 7-15 | 16-25 | 26-35 | 36-45 | >46 |
| *Maynas* | 298 | 143 | 155 | 72 | 83 | 42 | 48 | 53 |
| *Tupac Amaru* | 284 | 120 | 164 | 68 | 75 | 53 | 32 | 56 |
| Total | 582 | 263 | 319 | 140 | 158 | 95 | 80 | 109 |
|  |  |  |  |  |  |  |  |  |

**Table S2.** Maximum likelihood estimates of the probability of movement outside an individual’s home (*P(Δd)*). The data was better fitted by an exponential function of the form $P\left( \Delta d \right)= \varphi\cdot{exp}^{\tau\cdot\Delta d}+ \varepsilon\cdot{exp}^{\theta\cdot\Delta d} .$Table shows mean parameter values and 95% confidence intervals (CI). In all cases, R^2^ values of each fit were >0.9.

|  |  |  |  |  |  |
| --- | --- | --- | --- | --- | --- |
|  | Coefficients (95% CI) | | | |  |
| Age group | **φ** | **Τ** | **ε** | **Θ** |  |
| All ages | 0.8103 (0.8102, 0.8104) | -0.0432 (-0.04322, -0.04319) | 0.3183 (0.3182, 0.3184) | -5.17 E-04 (-5.17E-04, -5.17E-04) |  |
| 7-15 yrs | 0.878 (0.8777, 0.8783) | -0.04208 (-0.04211, -0.04205) | 0.2558 (0.2556, 0.2560) | -6.20E-04 (-6.22E-04, -6.19E-04) |  |
| 16-25 yrs | 0.8718 (0.8716, 0.872) | -0.04359 (-0.04362, -0.04357) | 0.2627 (0.2625, 0.2628) | -5.07E-04 (-5.08E-04, -5.07E-04) |  |
| 26-35 yrs | 0.3598 (0.3596, 0.3599) | -0.0004653 (-0.00046, -0.00047) | 0.763 (0.7627, 0.7632) | -0.04321 (-0.04324, -0.04318) |  |
| 36-45 yrs | 0.7289 (0.7287, 0.7291) | -0.03932 (-0.03934, -0.0393) | 0.3751 (0.3749, 0.3752) | -4.38E-04 (-4.38E-04, -4.37E-04) |  |
| > 45 yrs | 0.7954 (0.7952, 0.7956) | -0.04558 (-0.0456, -0.04555) | 0.3435 (0.3434, 0.3436) | -5.88E-04 (-5.89E-04, -5.88E-04) |  |

**Table S3.** Maximum likelihood estimates of the probability of visiting *y* places (*P(y)*). The data was best fit by a Weibull function of the form $\left( y \right)= a\times b\times y^{b-1}\times e^{(-a\times y^{b})}$ . The table shows mean parameter values and their standard error. In all cases, R^2^ values of each fit were >0.9.

|  | Coefficients (Standard Error) | | | |
| --- | --- | --- | --- | --- |
| Age group | Shape | | Scale | |
| All ages | 1.716963 | (0.053562) | 6.546896 | (0.167197) |
| 7-15 yrs | 1.676272 | (0.102385) | 5.834692 | (0.310647) |
| 16-25 yrs | 1.70958 | (0.102783) | 6.308605 | (0.310762) |
| 26-35 yrs | 1.677471 | (0.129791) | 6.362414 | (0.411718) |
| 36-45 yrs | 1.826416 | (0.162453) | 7.356161 | (0.473726) |
| >46 yrs | 1.805423 | (0.128992) | 7.322955 | (0.410845) |

**Table S4.** Fitted estimates of the degree distribution of *N_P_* and *N_L_*. The data was better fitted by a truncated power law of the form $P\left( k \right) \propto\left\{ \begin{aligned} ae^{-\lambda k} , k<k_{min} \\ k^{-\alpha} , k\geq k_{min} \end{aligned} \right.$(Kolmogorov-Smirnov, *P* > 0.1). The table shows mean parameter values and their standard error (in parenthesis).

|  | Exponential fit | |  | Power-law fit | |
| --- | --- | --- | --- | --- | --- |
| Network* | A | λ |  | kmin | α |
| NP | 1.48 (0.13) | -0.57 (0.06) |  | 5.0 (1.4) | 2.60 (0.10) |
| NL | 1.38 (0.03) | -0.16 (0.05) |  | 14.9 (1.6) | 3.24 (0.14) |
| * NP represents the affiliation network linking individuals who share at least one location, whereas NL represents the affiliation network linking locations that have at least one visitor in common. | | | | | |

**Table S5**. Parameter structure and default values for the individual based model linking human movement and the transmission of a directly transmitted pathogen.

| Parameters | Value* | |
| --- | --- | --- |
| **Model setup** |  | |
| Individuals | | 1000 |
| Houses | | 3000 |
| Work place | | 630 |
| Commercial place | | 270 |
| House operation time (hours) | | 24 |
| Work place operation time (hours) | | 12 |
| Commercial place operation time (hours) | | 16 |
| **Transmission module** | |  |
| Contact probability per time step (*Ci*) | | 0.2 / Number of co-located individuals |
| Probability of transmission | | 0.5**Ci* |
| Incubation period | | 2 days |
| Infectious period | | 4 days |
| Time step of simulation | | 15 min (105 days total) |
| **Iteration settings** | |  |
| Number of visited location per day per person | | WB: scale= 6.5, shape=1.7 |
| Distribution of link per location | | PL: γ=2.8 |
| Number of hours spent in a single location | | EXP: μ=1h; μ=2h; μ=3h; μ=4h |
| Number of time spent in traveling between location | | UNI: 15-120 min; step 15 min |
|  | |  |

* Where N represents total number of people at a location; WB, Weibull distribution; PL, Power-Law distribution; EXP, exponential distribution; UNI, uniform distribution.
