# Supplementary figures and images for: Using GPS Technology to Quantify Human Mobility, Dynamic Contacts and Infectious Disease Dynamics in a Resource-Poor Urban Environment
Source: PLoS One. 2013 Apr 8;8(4):e58802. doi: 10.1371/journal.pone.0058802 (PMC3620113; doi:10.1371/journal.pone.0058802)

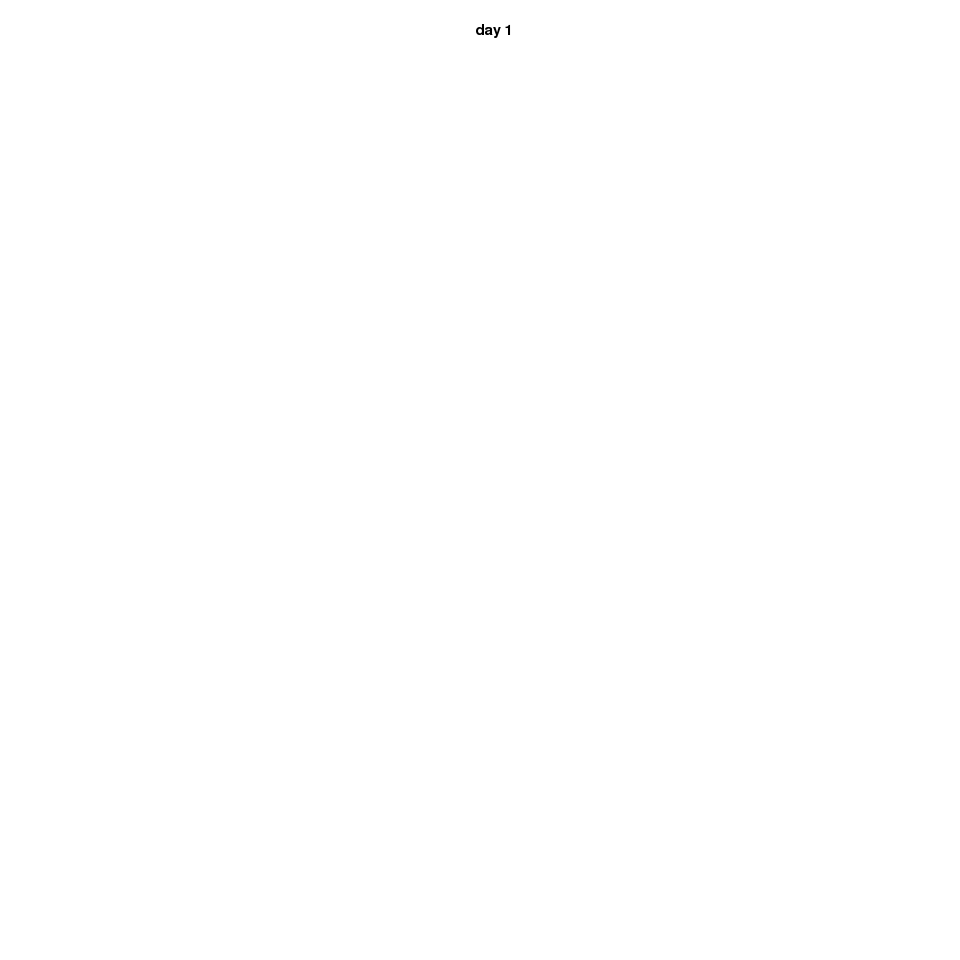

Supplement: File S3 — Supplementary Videos. Dynamic representation of the pathway followed by the introduced infection through the modeled population. Each video represent the temporal sequence of the infection when μ = 1 (Video S1), μ = 2 (Video S2), μ = 3 (Video S3) and μ = 4 (Video S4). (ZIP) [file pone.0058802.s003.zip › Video S1.gif]

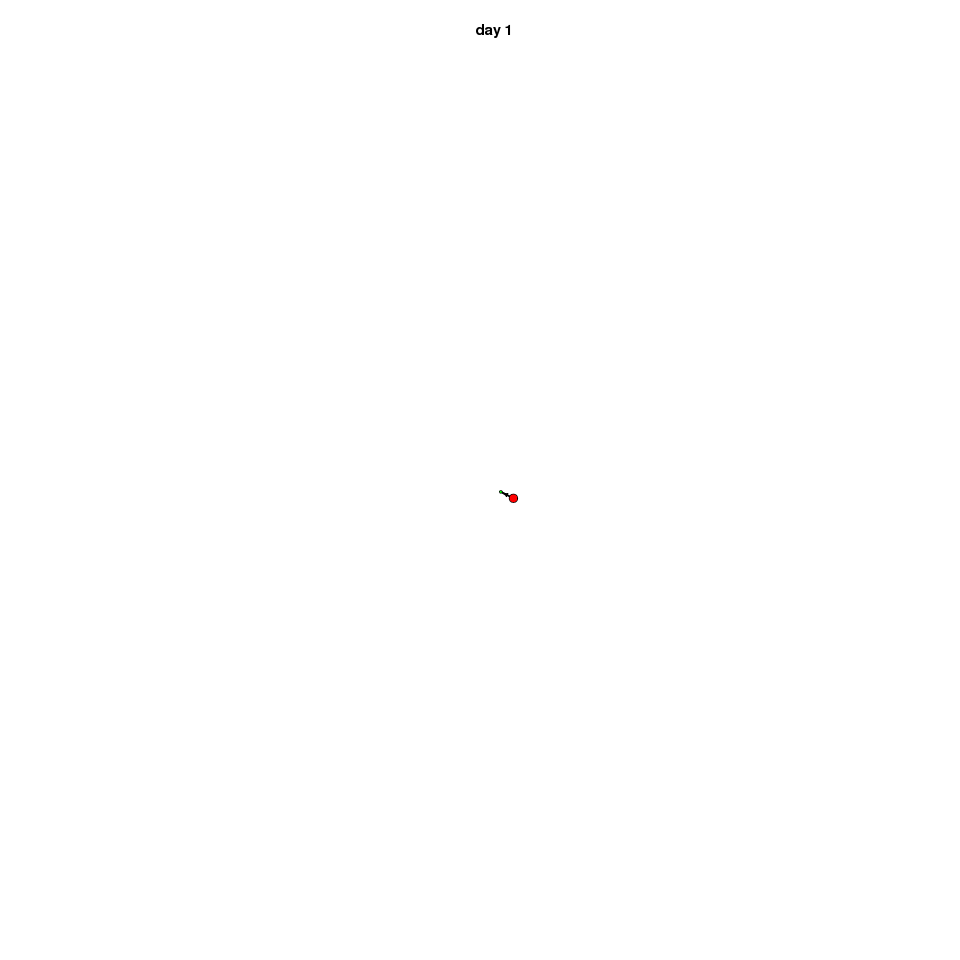

Supplement: File S3 — Supplementary Videos. Dynamic representation of the pathway followed by the introduced infection through the modeled population. Each video represent the temporal sequence of the infection when μ = 1 (Video S1), μ = 2 (Video S2), μ = 3 (Video S3) and μ = 4 (Video S4). (ZIP) [file pone.0058802.s003.zip › Video S2.gif]

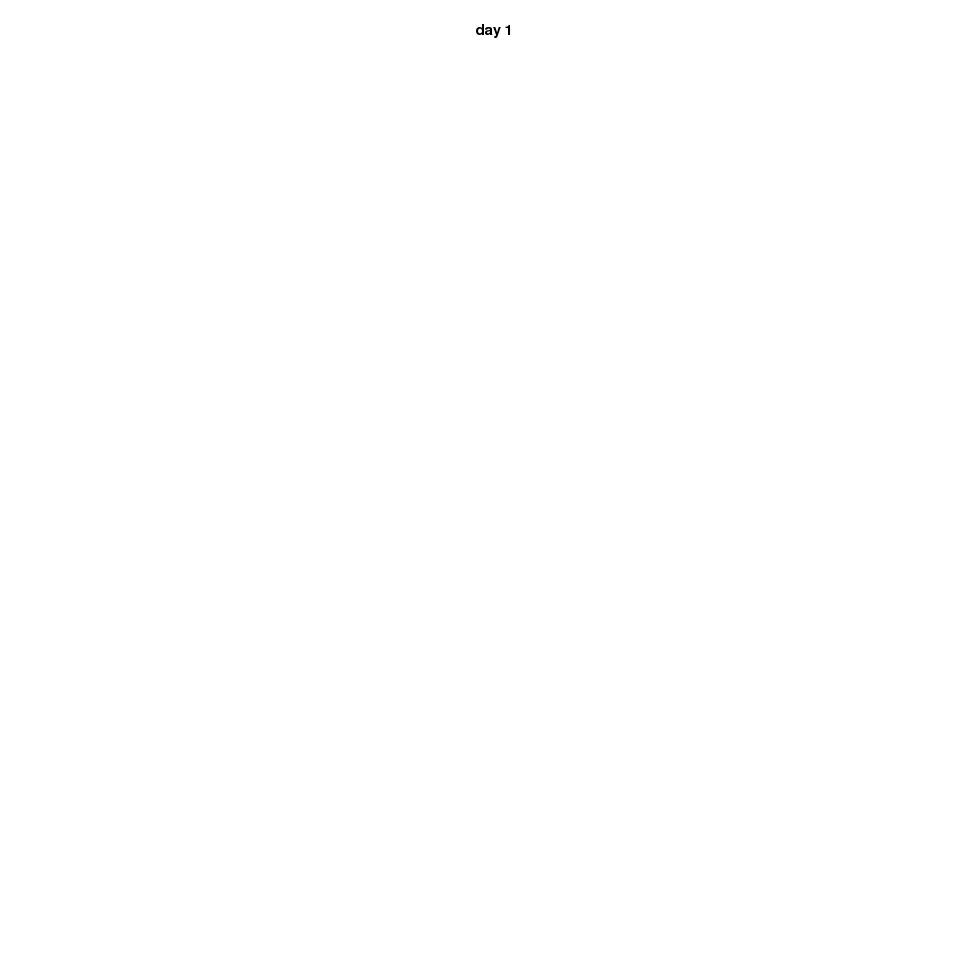

Supplement: File S3 — Supplementary Videos. Dynamic representation of the pathway followed by the introduced infection through the modeled population. Each video represent the temporal sequence of the infection when μ = 1 (Video S1), μ = 2 (Video S2), μ = 3 (Video S3) and μ = 4 (Video S4). (ZIP) [file pone.0058802.s003.zip › Video S3.gif]

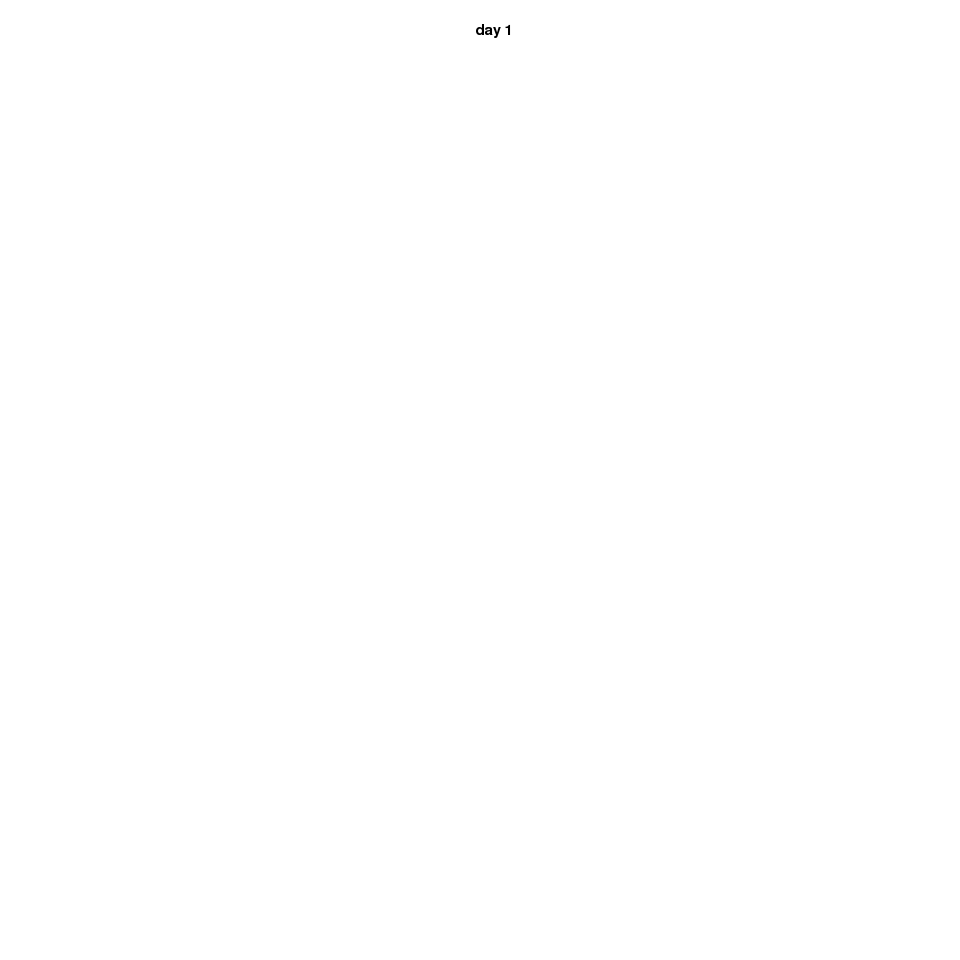

Supplement: File S3 — Supplementary Videos. Dynamic representation of the pathway followed by the introduced infection through the modeled population. Each video represent the temporal sequence of the infection when μ = 1 (Video S1), μ = 2 (Video S2), μ = 3 (Video S3) and μ = 4 (Video S4). (ZIP) [file pone.0058802.s003.zip › Video S4.gif]
